# Effect of temperature, CO_2_ and O_2_ on motility and mobility of Anisakidae larvae

**DOI:** 10.1038/s41598-021-83505-5

**Published:** 2021-02-19

**Authors:** Aiyan Guan, Inge Van Damme, Frank Devlieghere, Sarah Gabriël

**Affiliations:** 1 FMFP-UGent, Research Unit Laboratory of Food Microbiology and Food Preservation, Department of Food Technology, Safety and Health, Faculty of Bioscience Engineering, Ghent University, Coupure Links 653, 9000 Gent, Belgium; 2 Laboratory of Foodborne Parasitic Zoonoses, Department of Veterinary Public Health and Food Safety, Faculty of Veterinary Medicine, Ghent University, Salisburylaan 133, 9820 Merelbeke, Belgium

**Keywords:** Parasite physiology, Applied microbiology

## Abstract

Anisakidae, marine nematodes, are underrecognized fish-borne zoonotic parasites. Studies on factors that could trigger parasites to actively migrate out of the fish are very limited. The objective of this study was to assess the impact of different environmental conditions (temperature, CO_2_ and O_2_) on larval motility (in situ movement) and mobility (migration) in vitro. Larvae were collected by candling or enzymatic digestion from infected fish, identified morphologically and confirmed molecularly. Individual larvae were transferred to a semi-solid Phosphate Buffered Saline agar, and subjected to different temperatures (6 ℃, 12 ℃, 22 ℃, 37 ℃) at air conditions. Moreover, different combinations of CO_2_ and O_2_ with N_2_ as filler were tested, at both 6 °C and 12 °C. Video recordings of larvae were translated into scores for larval motility and mobility. Results showed that temperature had significant influence on larval movements, with the highest motility and mobility observed at 22 ℃ for *Anisakis* spp. larvae and 37 ℃ for *Pseudoterranova* spp. larvae. During the first 10 min, the median migration of *Anisakis* spp. larvae was 10 cm at 22 ℃, and the median migration of *Pseudoterranova* spp. larvae was 3 cm at 37 ℃. Larval mobility was not significantly different under the different CO_2_ or O_2_ conditions at 6 °C and 12 ℃. It was concluded that temperature significantly facilitated larval movement with the optimum temperature being different for *Anisakis* spp. and *Pseudoterranova* spp., while CO_2_ and O_2_ did not on the short term. This should be further validated in parasite-infected/spiked fish fillets.

## Introduction

Marine ascaridoids, particularly of the family Anisakidae, are underrecognized zoonotic foodborne parasites which may have a substantial influence on public health. They lead to human anisakidosis^1–3^, which is mainly caused by *Anisakis simplex *sensu stricto*, Anisakis pegreffii* and *Pseudoterranova decipiens*^4–6^, while other members of the Anisakidae and Raphidascaridae family are less commonly responsible for human infections^7,8^. High prevalence rates of Anisakidae have been found in most wild commercial marine fish species (some species close to 100%), with a worldwide distribution^9,10^. The occurrence of Anisakidae varies between fish species and fishing sea. For example, Mercken et al. reported a mean prevalence of respectively 95% and 5% in Pollack and Atlantic salmon, with fish caught in the Northeast Atlantic having the highest infection rate (68%) from all investigated fishing zones^11^. Human infection after the consumption of fish infected with the L3 larvae may lead to a variety of symptoms, ranging from mild to severe abdominal pain and mild allergic reactions to anaphylaxis^7,12^. The latter is caused by specific allergens of the larvae, of which some are heat and freeze resistant^13^. An increase in fish and human cases has occurred in the past few decades^14^. Globally, the number of human cases was estimated to be 20,000 per year prior to 2010, with the majority attributable to Japan^15^. The consumption of sushi and sashimi (raw fish dishes) is an important source of human infection in Japan^14,16^, with 2000–3000 cases of anisakiasis being reported per year^17^. However, in a study of Bao and colleagues^18^, the number of cases needing hospitalization in Spain was estimated to be 8,320 per year, excluding mild infections, which indicates that the earlier mentioned global figures are probably seriously underestimated^18^. In addition to the impact on public health, these parasites are also an important cause of economic cost for the sector. Because of the public health risk, the European Union Regulation No 853/2004 stipulates that all fish destined for raw consumption, cold-smoking processing with internal temperature below 60 ℃, marinated or salted process should be frozen at a temperature of no higher than -20 ℃ for at least 24 h^19^. This leads to an important cost for the industry and has consequences on the quality of the fish. In addition, the sale of fresh fish has led to a growing number of consumer complaints, because the larva can become very mobile and therefore very visible for the consumer on top of the fillets^9^. The problem was reported to be more pronounced with the introduction of modified atmosphere packaging (MAP). This packaging system, including a mixture of oxygen (O_2_), carbon dioxide (CO_2_) and nitrogen (N_2_) in the headspace of a package, guarantees the extension of the shelf life of fresh products and is intensively applied by the fish industry to present packaged fresh fish to the consumer at the retail level. However, increased carbon dioxide levels have been reported to activate parasitic larvae^20^, resulting in more consumer complaints, which have a negative impact on the sales and therefore sector.

Recent risk models showed that inactivating or removing infectious larvae would greatly reduce the number of human cases^18^. The most commonly used techniques to inactivate larvae, such as freezing^21,22^ or heating^23^, also change the organoleptic properties of the fish. Moreover, the fish is no longer considered as “fresh” and the effects of certain allergens may still exist^12^, so the allergic public health risk persists. The fish industry tries to identify infected fish / fish fillets and remove the larvae manually in the case of light infections, while heavily infected fish are being discarded. The routinely used diagnostic method is candling, by which the fish fillets are placed on a transparent plate above a light source. Visible larvae can be removed from the fillets during this process. However, this technique is very labor intensive and lacks sensitivity^24^, thereby neither eliminating the public health risks, nor consumers' complaints.

An applicable method which can remove larvae quickly from infected fish, without affecting its quality and without increasing the microbiological contamination should therefore be developed. Currently, there is no such technique available. To allow the development of such a method, more insight is necessary in the triggers for larvae to actively migrate out of the fish flesh. According to Simat et al.^25^, certain biochemical changes in dead fish trigger *post mortem* migration of larvae from viscera to flesh. Examples of such biochemical changes are the production of biogenic amine^25^, the accumulation of fatty acid, lactic acid or phosphoric acid, which decrease the pH of flesh^26,27^. But those factors are not implementable in practice. As mentioned above, Pascual et al. studied larval motility, mobility and behavior after 3 days of storage and found that MAP (CO_2_, O_2_) affected the migration of larvae^20^. Cipriani et al. studied larval migration after storage at low temperature (2 ℃, 5 ℃, 7 ℃) for 24 h, 48 h and 72 h, concluding that temperature plays an important role in larval *post mortem* mobility^26^. Moreover, there is an association between temperature and gene expression levels of some antigenic and functional proteins released by zoonotic species of *Anisakis*, which may be involved in the host tissue migration of the parasite in the hostile target tissues of the fish host^28^. Long treatment periods are unsuitable for the fish processing industry considering the short shelf-life of fresh fish. There are no studies that have systematically evaluated short-term effects (in a timeframe useful for the industry) of temperature, CO_2_ and O_2_ on the motility/mobility of larvae. The objectives of this study were therefore to investigate the short-term effects of temperature, CO_2_ and O_2_ on larval motility and mobility. For this, *Pseudoterranova* spp. larvae and *Anisakis* spp. larvae were subjected to different conditions of temperature, CO_2_ and O_2_ with N_2_ as filler in vitro. Larval movements were video recorded, transcribed and analyzed. The study will provide information about the potential of the above-mentioned factors to trigger larvae to actively move, which could then potentially be applied to quickly remove larvae from fish in an industrial setting.

## Materials and methods

### Collection of larvae

Fish and larvae (the latter collected during industrial candling) were supplied by a fish whole-sale company in Belgium and transported to the laboratory on ice on a weekly basis until the end of the experiment. Fish (mainly mackerel, blue whiting, cod, herring and pollack^29^) were skinned and filleted upon arrival and digested the following day to collect the Anisakidae larvae, which were subsequently defined as one batch. Anisakidae larvae collected during the industrial candling were rinsed and cleaned, and digested if too much fish tissue was present, and such collected larvae were defined as another batch. Digestion was done as described by Jackson et al. with a minor modification^30^. Briefly, fish muscle and viscera were separately put in a pepsin and HCl digestion solution at 37 ℃ and stirred for 30 min. The digested fish solution was poured over a sieve where after the contents of the sieve were rinsed into a petri dish placed on the candling table. Larvae were collected, visually assessed for integrity and viability and identified according to the macroscopic morphological features of larvae^31,32^ into macroscopically assumed *Pseudoterranova* spp. and *Anisakis* spp.. After the in vitro experiment, subsets of larvae were identified molecularly (see 2.5). Larvae from the same batch were allocated to each of the conditions under evaluation (see 2.3), so that larvae from the same source were used for pairwise comparison of different conditions. Grouped larvae were stored in Phosphate Buffered Saline (PBS) at 4 °C until use within 24 h.

### In vitro experimental setup

#### Medium preparation

To observe larval movement under different temperature, CO_2_ and O_2_ conditions, Phosphate Buffered Saline (PBS) agar medium was prepared as a carrier. To determine the optimal agar concentration for this study, the migration and ease of visual inspection of larvae in 2, 2.5, 3, 3.5 g/L agar were compared in a preliminary experiment. Finally, 2.5 g/L of agar was chosen as the most optimal concentration as larvae could actively move or coil in 2.5 g/L agar and PBS medium and were easily visible.

Firstly, 1000 mL PBS solution was prepared using 5 PBS tablets (Sigma-Aldrich, St. Louis, MO, USA), then 2.5 g agar was added. The solution was mixed and dissolved by heating with frequent agitation. Lastly the solution was boiled for 1 min and transferred to a water bath at 45 °C to cool down for further use.

#### Preparation of larvae within the medium

**Figure 1:**
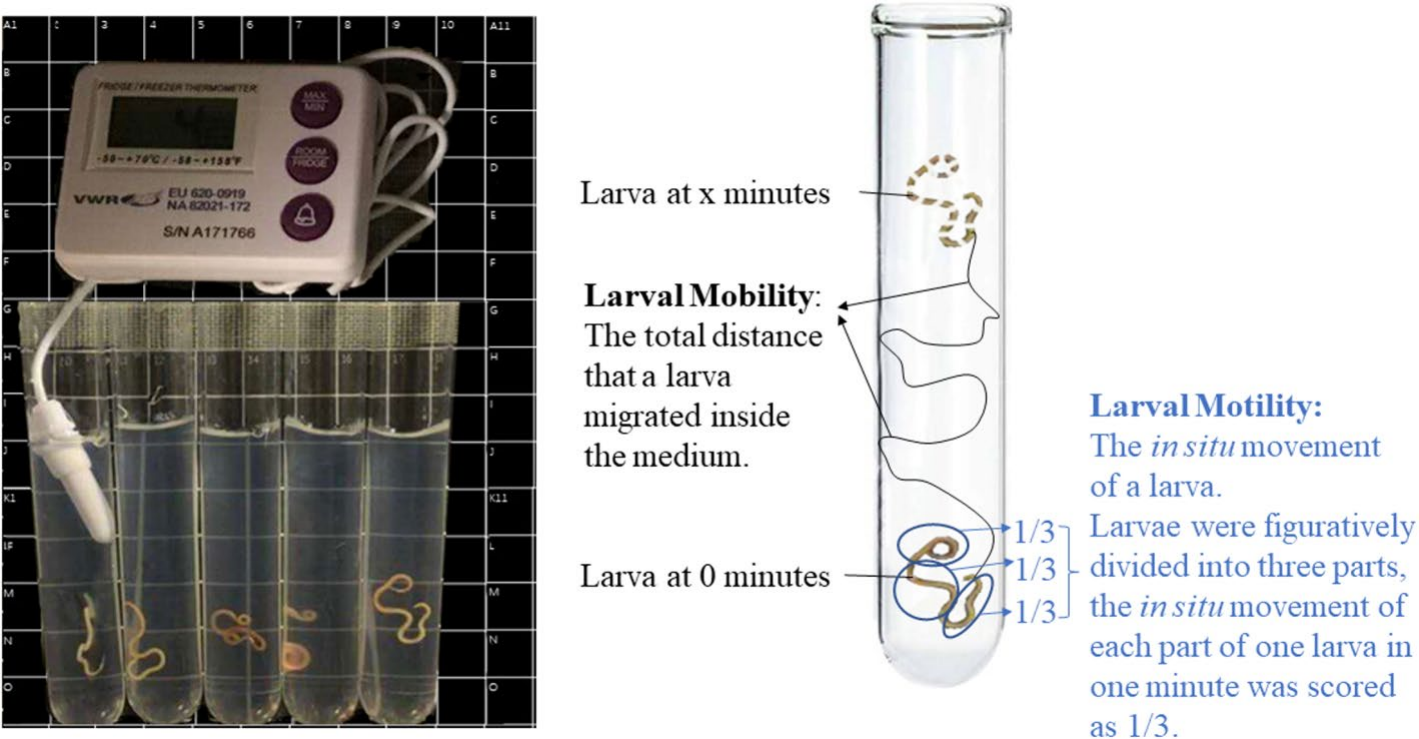
Experimental setup (left) and measurements of larval movements (right).

Tubes and larvae were placed at 4 ℃ to cool down for 10 min. One mL of medium was poured into each tube and solidified immediately. Then, one larva was transferred in each of the tubes, after which another 9 mL of the medium was poured into each tube. The tubes were then placed in the fridge at 4 °C to ensure a rapid solidification of the agar medium. The temperature of the medium inside the tubes was monitored by a thermometer. Once the temperature of the medium reached 4 ℃, the tubes were ready for further use. Five tubes from which one contained a thermometer were positioned in front of a black background as visualized in Fig. 1. This setup was repeated for a total of 4 times per condition, for both *Pseudoterranova* spp. and *Anisakis* spp., resulting in a total of 20 replicates per condition and per genus, except that 21 larvae were investigated for the effect of temperature on *Anisakis* spp. larvae and 22 larvae were investigated for the effect of CO_2_ on *Pseudoterranova* spp. larvae at 6 ℃.

### Larval treatments at different temperatures and gas atmospheres

To evaluate the effect of temperature, 6 ℃, 12 ℃, 22 ℃ and 37 ℃ were chosen based on previous studies^26^ on the one hand, as those temperature ranges may contribute to increasing the movement of larvae and on the other hand, in a more arbitrary way, aiming to cover a sufficiently wide temperature range including 37 ℃ which approaches the temperature of marine mammals, the final hosts of larvae. The tubes with background were put in a ventilated refrigerator or incubator at respectively 6 ℃, 12 ℃, 22 ℃ and 37 ℃ with a timer and thermometer recording the time and real-time medium temperature. Larval movements were registered by video recording using a tablet (Apple, A1822, USA) with a light source. After 30 min, the recording was stopped.

For the CO_2_ and O_2_ experiment, modified atmosphere packaging was applied. Different CO_2_ and O_2_ concentrations were evaluated at two different temperatures, 6 °C and 12 °C. Tubes with larvae, background and thermometer were placed in a plastic packaging tray (Decapac, HERENTALS, Belgium) and transferred to the fridge at 6 or 12 ℃. When the temperature of the medium reached the destinated temperature, silica gel (Type II, 3.5 mm bead size) (Sigma-Aldrich, St. Louis, MO, USA) was put into the tray to eliminate the moisture outside the tubes and trays were immediately modified atmosphere packaged at the required gas mixture with a MAP tray sealer machine (Deca, GROSCHOPP, Germany). A top film (Bemis Packaging Benelux, MONCEAU-SUR-SAMBRE, Belgium) with the specifications OPALEN/HB/65/AF/PP/PL was used. For the CO_2_ experiment, 20%, 40%, 60%, 80%, or 90% CO_2_ was used for the experiment at 12 °C and 0%, 20%, 60% or 90% CO_2_ for the experiment at 6 °C. In all packages 5% O_2_ was added together with N_2_ as a compensation gas. For the O_2_ experiment at 6 and 12 ℃, 0%, 5%, 21% or 80% O_2_ was used compensated with N_2_. After packaging, trays were put back into the corresponding fridge with a timer recording the time. Larval movements were registered by video recording during the first 30 min. Additionally, for the experiments at 6 ℃, the movements were further recorded during 1-min recordings every 30 min for 6 h.

### Assessment of larval movements

Larval movements were assessed through video recording (see “Larval treatments at different temperatures and gas atmospheres”). The video recordings were analyzed to define larval motility and mobility to evaluate the changes of larval movements, which is shown in Fig. 1.

#### Assessment of larval motility

Larval motility was defined as the in situ movement of a larva. To quantify this motility, larvae were figuratively divided into three parts: the head part, the middle part and the tail part. In 1 min, the in situ movement of each part of one larva was scored as 1/3. For example, if only one part of the larva moved in 1 min, the motility of this larva is 1/3; if two body parts moved, the motility of larva for that minute is 2/3; if all three parts of the larva moved in 1 min, the motility of the larva was recorded as 1. The motility of larvae was determined for each video recorded minute. For the 30-min movies, the motility of one larva in 30 min was calculated as the sum of larval motility of each minute.

#### Assessment of larval mobility

Larval mobility is defined as the total distance that a larva migrated inside the medium. To determine the migration distance of larvae, the tubes were placed on a black background with white lines; the distance between each line was 1 cm. For larvae that had moved out of the medium, their migration distance inside the medium was divided by the time during which larvae were inside the medium and the value was multiplied by 30. For the effect of temperature, larval mobility during the first five and ten minutes was also analyzed. Similar as for 30 min, for larvae that had moved out of the medium during the first five or ten minutes, the migration distance inside the medium was divided by the time during which larvae were inside the medium in the first five or ten minutes and the value was multiplied by five or ten, respectively.

### Identification of larvae at species level

After the movies were taken, larvae were stored in ethanol for a subgroup identification by Polymerase Chain Reaction combined with Restriction Fragment Length Polymorphism (PCR/RFLP). For each tested condition, four larvae were selected for *Anisakis* spp. identification (one larva from each tray) and one larva for *Pseudoterranova* spp. identification. In total, 110 larvae were selected and identified. DNA extraction from larvae and PCR amplification for the Internal Transcribed Spacer (ITS) fragment were implemented according to Zhu et al*.*^33^. The amplified ITS fragments were digested by endonucleases *HinfI* and *HhaI* following the protocol from the European Reference Laboratory (ISS, 2018). The PCR/RFLP profiles were confirmed, followed by a species identification of larvae^34–36^.

### Statistical analysis

Video recordings were transcribed into Excel (Microsoft office 2016, Microsoft, USA) according to the definition of assessment of larval movements (motility and mobility). In total, 11 larvae were excluded from the analyses because they were dead (n = 2) or because they were on top of the medium at the beginning of the experiment (n = 9). Conditions were only compared within experiments that used larvae from the same batch(es) to account for a potential batch effect. Since the data were not normally distributed, non-parametric Kruskal–Wallis rank sum tests were carried out to check if the movements were the same for each of the conditions. If at least one condition differed (p < 0.05), pairwise comparisons between conditions were performed using Wilcoxon rank sum tests (= Mann–Whitney tests) with Bonferroni corrections for multiple testing. R version 3.6.2 was used for all statistical analyses (R Core Team, 2019).

## Results

### Larvae identification

After the preliminary morphological identification of larvae, molecular analysis was conducted on respectively a total of 88 and 22 macroscopically assumed *Anisakis* spp. and *Pseudoterranova* spp. larvae. For *Anisakis* spp., 86 larvae were identified as *A. simplex*, and two as *A. pegreffii*. The latter two larvae were both from the CO_2_ experiment, one from 0% CO_2_ at 6 ℃ and the other from 90% CO_2_ at 12 ℃. All *Pseudoterranova* spp. larvae were identified as *P. decipiens* s.s.

### Effect of temperature on larval movements

**Figure 2:**
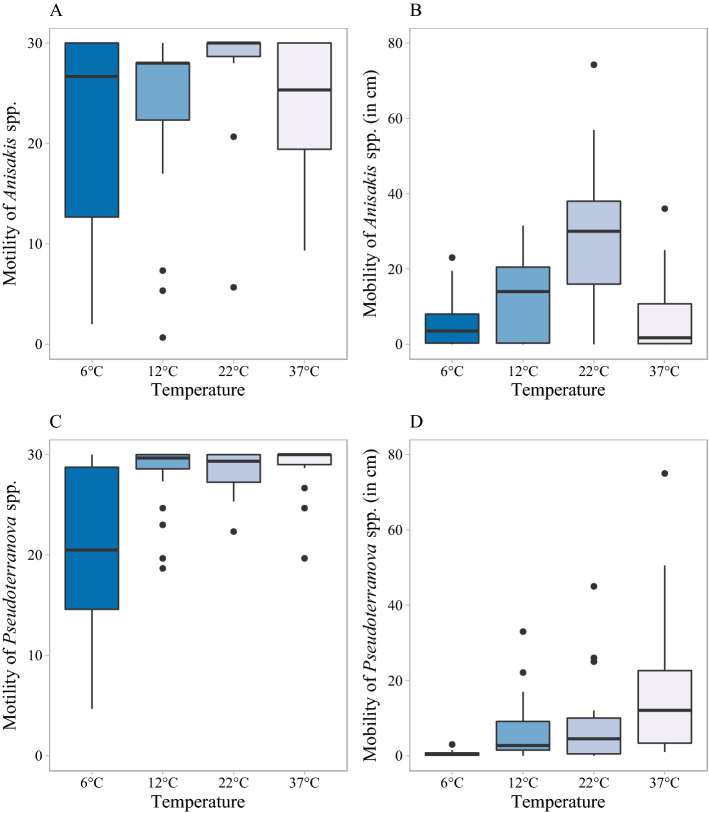
Effect of temperature on larval motility and mobility (in cm) during the first 30 min. **(A)** Motility of *Anisakis* spp., **(B)** mobility of *Anisakis* spp., **(C)** motility of *Pseudoterranova* spp. and **(D)** mobility of *Pseudoterranova* spp. under different temperature conditions. The box consists of the upper quartile, median and lower quartile. The whiskers are drawn up to the highest or lowest observed point from the dataset that falls within 1.5 times the interquartile range. Observations beyond the end of the whiskers are plotted individually.

The effects of four different temperatures on larval motility and mobility are shown in Fig. 2. For *Anisakis* spp., temperature had a significant effect on larval motility (Kruskal–Wallis test: χ^2^-value = 11.1, df = 3, p = 0.011), with the highest motility at 22 °C. At this temperature, the median value for motility was 30, which indicates that over 50% of larvae were moving from head to tail during the full 30 min. During the 30 min, the motility of *Anisakis* spp. larvae at 22 °C was significantly higher compared to larval motility at 12 °C (p = 0.007). All *Anisakis* spp. larvae were motile in at least 1 min of the 30 min at all temperatures, but there was a variation between larvae. For example, 7 out of 21 *Anisakis* spp. larvae were motile during the full 30 min at 6 ℃, but there were also 3 *Anisakis* spp. larvae that had a motility less than 5.

There were respectively 9 out of 21 and 3 out of 20 *Anisakis* spp. larvae that migrated out of the medium at 22 ℃ and 37 ℃. Similar as the motility of *Anisakis* spp. larvae, also the mobility of *Anisakis* spp. larvae differed at the four temperatures (χ^2^ = 22.6, df = 3, p < 0.001). Mobility of *Anisakis* spp. larvae was significantly higher at 22 ℃ compared to the mobility at other temperatures (p ≤ 0.02). At 22 ℃, the median migration of *Anisakis* spp. larvae was 30 cm, but mobility varied greatly between the different larvae, ranging between 0 and 74 cm (interquartile range, IQR = 22). Only one out of 21 *Anisakis* spp. larvae didn’t migrate at 22 °C, while 5 *Anisakis* spp. larvae didn’t migrate at 6 °C and 37 °C (Table 1). Mobility of *Anisakis* spp. larvae at 6 ℃ was significantly lower compared with larval mobility at 22 ℃ (W = 54.5; p < 0.001). At 6 °C, *Anisakis* spp. larvae migrated between 0 and 23 cm (IQR = 7.7), with a median migration of 3.5 cm. At 12 ℃, *Anisakis* spp. larvae had a median migration of 14 cm. Mobility of *Anisakis* spp. larvae at 37 ℃ decreased significantly compared with larval mobility at 22 ℃ (W = 356.5; p < 0.001). At 37 °C, *Anisakis* spp. larvae had a median migration of 1.7 cm (range between 0 and 36 cm; IQR = 10.6).

**Table 1:** Number of larvae that were motile/mobile in 30 min of CO_2_ and O_2_ experiments.

Parasite	Category	Temperature				CO_2_ at 6 ℃				CO_2_ at 12 ℃					O_2_ at 6 ℃				O_2_ at 12 ℃			
		6 ℃	12 ℃	22 ℃	37 ℃	0%	20%	60%	90%	20%	40%	60%	80%	90%	0%	5%	21%	80%	0%	5%	21%	80%
*Anisakis* spp. larvae	MT	21	21	21	20	20	19	19	19	15	19	18	18	15	15	17	16	8	12	12	13	10
	MB	16	18	20	15	10	9	7	9	9	14	12	11	8	4	8	11	1	3	6	5	2
	N	21	21	21	20	20	20	19	20	18	19	19	20	19	20	20	20	20	20	20	20	20
*Pseudoterra-nova* spp. larvae	MT	20	20	20	18	17	21	22	17	20	19	20	20	20	20	20	20	20	20	20	20	19
	MB	15	19	18	18	5	13	5	8	18	19	19	19	20	12	12	8	8	14	10	16	12
	N	20	20	20	18	17	22	22	17	20	19	20	20	20	20	20	20	20	20	20	20	19

*MT* the number of larvae that were motile in 30 min, *MB* the number of larvae that were mobile in 30 min, *N* total number of larvae analyzed (excluding ineligible larvae).

Larval mobility under four different temperatures was also evaluated respectively during the first 5 and 10 min. During the first 10 min, mobility of *Anisakis* spp. larvae at 22 ℃ was already significantly higher than the mobility at other temperatures (p < 0.01). At 22 ℃, *Anisakis* spp. larvae had a median migration of 10 cm and a maximum value of 30 cm in 10 min. Also, 20 out of 21 *Anisakis* spp. larvae (95%) were mobile at 22 ℃ while only 59% to 67% of *Anisakis* spp. larvae were mobile at the other temperatures during the first 10 min. During the first 5 min, the mobility of *Anisakis* spp. larvae at 22 ℃ was already significantly higher than the mobility of *Anisakis* spp. larvae at 6 ℃ and 12 ℃ (p < 0.01), but the difference in the mobility of *Anisakis* spp. larvae between 22 ℃ and 37 ℃ was not significant (p = 0.057). After 5 min, *Anisakis* spp. larvae had a median migration of respectively 3 cm and 0.7 cm at 22 ℃ and 37 ℃ and at 22 ℃ 86% of *Anisakis* spp. larvae were mobile while only 57% to 65% of *Anisakis* spp. larvae were mobile at the other temperatures.

All *Pseudoterranova* spp. larvae were motile during at least 1 min of the 30 min at all temperatures. Motility of *Pseudoterranova* spp. larvae at 6 ℃ was significantly lower than larval motility at other temperatures with p < 0.01 (Fig. 2). There was no significant difference in motility of *Pseudoterranova* spp. larvae between 12, 22 and 37 ℃ and there were respectively 40%, 40% and 56% *Pseudoterranova* spp. larvae that had a motility value of 30 at 12, 22 and 37 °C, meaning that those larvae were fully motile during every minute of the 30 min.

For the mobility of *Pseudoterranova* spp. larvae, the highest migration was observed at 37 ℃ (Fig. 2). There were 6 out of 18 *Pseudoterranova* spp. larvae that migrated out of the medium at 37 ℃. All *Pseudoterranova* spp. larvae were mobile at 37 °C, with a median migration of 12 cm, and a maximum of 75 cm. Mobility of *Pseudoterranova* spp. larvae at 6 °C ranged between 0 and 3 cm and was significantly lower compared to mobility at 12 ℃ (W = 39; p-value < 0.001), 22 ℃ (W = 84.5; p-value = 0.01) and 37 ℃ (W = 8.5; p-value < 0.001). The differences of larval mobility between 12, 22 and 37 ℃ were not significant (p > 0.05). There were five *Pseudoterranova* spp. larvae that did not migrate at 6 ℃ with all *Pseudoterranova* spp. larvae having a mobility at 37 ℃ (Table 1). The same trend was already observed after 5 and 10 min, with the lowest mobility at 6 °C compared with other temperatures (p < 0.01). During the first 10 min, *Pseudoterranova* spp. larvae had a median migration of 3 cm at 37 ℃, with a maximum of 26 cm. Moreover, 90% of *Pseudoterranova* spp. larvae were mobile at 37 ℃ during the first 10 min, while 70% were mobile at 12 and 22 ℃ and only 30% were mobile at 6 ℃. During the first 5 min, *Pseudoterranova* spp. larvae had a median migration of 1.5 cm at 37 ℃, with a maximum of 11 cm. During the first 5 min, 83% of *Pseudoterranova* spp. larvae were mobile at 37 ℃, while only 65% and 60% of *Pseudoterranova* spp. larvae were mobile at 12 and 22 ℃ and 20% of *Pseudoterranova* spp. larvae were mobile at 6 ℃

### Effect of CO_2_ on larval movements

The effect of different CO_2_ concentrations on larval movement was tested at 6 °C and at 12 °C. At 6 ℃, as shown in Table 1, more than 95% of *Anisakis* spp. larvae showed a level of motility for each condition, but only 37% (at 60% CO_2_) to 50% (at 0% CO_2_) of *Anisakis* spp. larvae were mobile (see Supplementary Fig. 1). At 6 ℃, there was no significant difference in the motility or mobility of *Anisakis* spp. larvae between the different tested CO_2_ concentrations. The mobile larvae only migrated a very short distance with more than 96% of *Anisakis* spp. larvae having a mobility below 2.5 cm.

**Figure 3:**
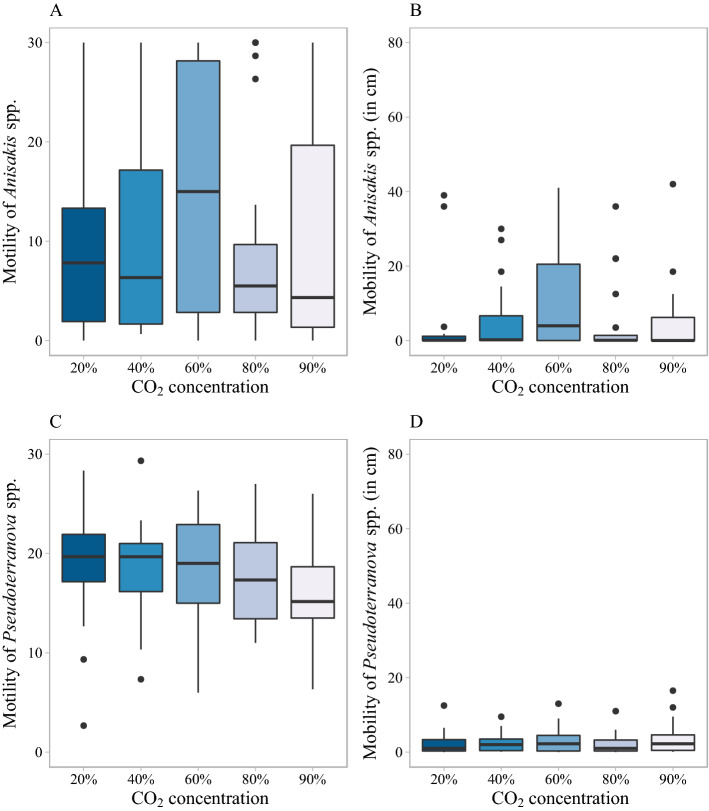
Effect of CO_2_ on larval motility and mobility at 12 ℃ during the first 30 min. **(A)** Motility of *Anisakis* spp., **(B)** mobility of *Anisakis* spp., **(C)** motility of *Pseudoterranova* spp. and **(D)** mobility of *Pseudoterranova* spp. under different CO_2_ conditions. The box consists of the upper quartile, median and lower quartile. The whiskers are drawn up to the highest or lowest observed point from the dataset that falls within 1.5 times the interquartile range. Observations beyond the end of the whiskers are plotted individually.

At 12 °C, as it is shown in Table 1, most *Anisakis* spp. larvae were motile under the different CO_2_ concentrations. The motility and mobility of *Anisakis* spp. larvae under 20%, 40%, 60%, 80% and 90% CO_2_ at 12 ℃ are shown in Fig. 3. There was no significant difference of larval motility for *Anisakis* spp. larvae at different CO_2_ concentrations at 12 °C, but the highest median motility (15) was observed at 60% CO_2_. Depending on the condition, between 30 to 50% of *Anisakis* spp. larvae did not migrate at 12 ℃, the median larval mobility was therefore very close to 0 at 20%, 40%, 80%, 90% CO_2_. Similar as for larval motility, *Anisakis* spp. larvae had the highest median (4 cm) at 60% CO_2_, but there were no significant differences of larval mobility between different CO_2_ concentrations for *Anisakis* spp. larvae at 12 °C.

**Figure 4:**
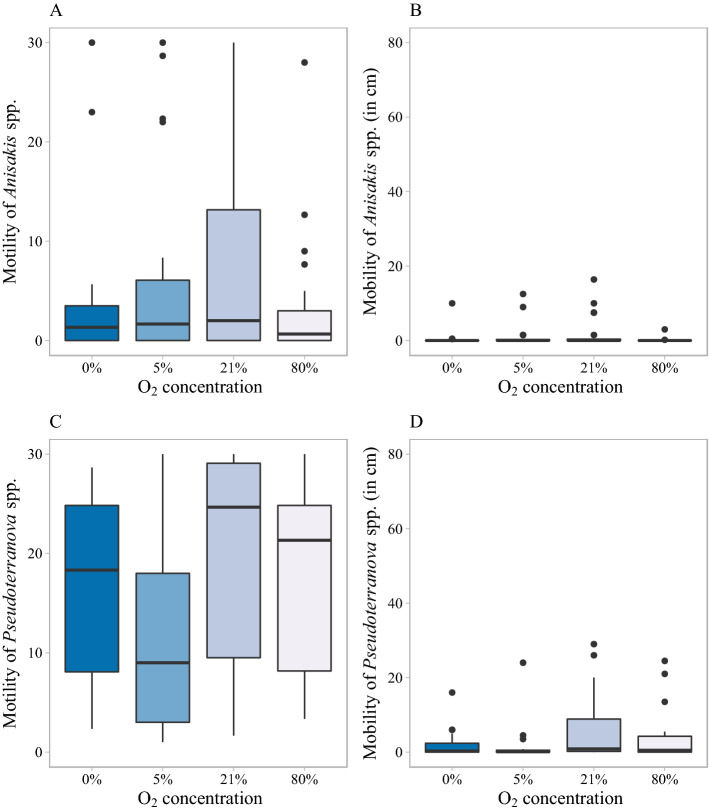
Effect of O_2_ on larval motility and mobility at 12 ℃ during the first 30 min. **(A)** Motility of *Anisakis* spp., **(B)** mobility of *Anisakis* spp., **(C)** motility of *Pseudoterranova* spp. and **(D)** mobility of *Pseudoterranova* spp. under different O_2_ conditions. The box consists of the upper quartile, median and lower quartile. The whiskers are drawn up to the highest or lowest observed point from the dataset that falls within 1.5 times the interquartile range. Observations beyond the end of the whiskers are plotted individually.

For *Pseudoterranova* spp. at 6 ℃, only one larva at 20% CO_2_ was not motile, while all other *Pseudoterranova* spp. larvae were motile in at least 1 min of the total 30 min under all tested concentrations of CO_2_. The motility of *Pseudoterranova* spp. larvae at 20% CO_2_ was significantly higher compared to larval motility at 0% CO_2_ (p = 0.043) (see Supplementary Fig. 1). There were only 23% (60% CO_2_) to 59% (20% CO_2_) *Pseudoterranova* spp. larvae that were mobile at different CO_2_ concentration at 6 ℃ (Table 1) with a maximum migration of only 1.5 cm in 30 min. No significant differences in migration of *Pseudoterranova* spp. larvae between the different CO_2_ concentrations were observed at 6 ℃.

At 12 ℃ (Fig. 3), all *Pseudoterranova* spp. larvae were motile, with no significant differences in larval motility between different CO_2_ concentrations. More than 90% of *Pseudoterranova* spp. larvae were mobile under each CO_2_ condition, with the migration ranging between 0 and 16.5 cm, but similar as for motility, there were no significant differences in larval mobility between different CO_2_ concentrations.

For the effect of CO_2_ on larval movements measured every 30 min for 6 h at 6 ℃, there were only 1 *Anisakis* spp. larvae and 7 *Pseudoterranova* spp. larvae that were still mobile after 6 h. The number of larvae that are still motile every 30 min until 6 h at different CO_2_ conditions is shown in Table 2. For *Anisakis* spp. larvae, the number of larvae that were motile at 1 h decreased suddenly compared with the number of motile larvae at 0.5 h. More *Pseudoterranova* spp. larvae were still motile after 6 h compared with *Anisakis* spp. larvae at 0%, 20%, 60% and 90% CO_2_.

**Table 2:** Number of larvae that were motile in 1-min recordings every half hour during 6 h.

Parasite	Conditions	Time point (h) at which one-minute recording was performed within a total period of 6 h													N
		0	0.5	1	1.5	2	2.5	3	3.5	4	4.5	5	5.5	6	
*Anisakis* spp. larvae	0%CO_2_	16	16	4	3	5	4	2	5	3	3	3	0	2	20
	20%CO_2_	15	13	8	4	2	5	2	6	2	5	1	4	2	20
	60%CO_2_	12	7	2	2	1	5	1	0	2	0	1	1	3	19
	90%CO_2_	13	12	2	0	0	3	1	2	0	1	4	0	1	20
	0%O_2_	7	6	4	1	4	4	2	1	1	2	1	1	1	20
	5%O_2_	5	9	5	4	4	4	2	0	3	4	0	1	1	20
	21%O_2_	8	12	5	7	8	7	6	7	6	5	6	5	5	20
	80%O_2_	4	2	2	2	2	1	0	2	3	4	2	0	2	20
*Pseudoterranova* spp. larvae	0%CO_2_	12	10	9	7	11	5	8	10	8	8	10	9	6	17
	20%CO_2_	16	18	14	14	14	14	13	11	12	12	5	9	7	22
	60%CO_2_	10	13	18	12	16	12	8	14	9	12	14	9	8	22
	90%CO_2_	11	14	5	9	6	11	14	14	11	10	11	11	9	17
	0%O_2_	18	11	16	18	14	15	8	16	14	14	13	7	4	20
	5%O_2_	16	18	14	19	12	14	17	13	13	10	12	14	16	20
	21%O_2_	16	14	11	13	14	15	15	14	15	16	11	12	12	20
	80%O_2_	8	15	11	9	9	10	12	12	5	12	8	10	11	20

*N* total number of larvae analyzed (excluding ineligible larvae).

1-min recordings were taken every 30 min for 6 h at different conditions of CO_2_ and O_2_ experiments at 6 ℃.

### Effect of O_2_ on larval movements

Larval motility and mobility under 0%, 5%, 21% and 80% O_2_ were studied respectively at 6 ℃ (see Supplementary Fig. 2) and 12 ℃ (Fig. 4) in absence of CO_2_.

For *Anisakis* spp., at 6 ℃, as shown in Table 1, more than 75% of *Anisakis* spp. larvae were motile at 0%, 5% and 21% O_2_, whereas at 80% O_2_ only 8 out of 20 *Anisakis* spp. larvae were motile. The motility of *Anisakis* spp. larvae at 21% O_2_ was significantly higher than larval motility at 80% O_2_ (p = 0.011), and the motility of *Anisakis* spp. larvae at 80% O_2_ was also significantly lower than larval motility at 5% O_2_ with p = 0.031 (see Additional file 2). For the mobility of *Anisakis* spp. at 6 ℃, more *Anisakis* spp. larvae migrated at 21% O_2_ compared with other O_2_ concentrations (Table 1). Larval mobility at 21% O_2_ was higher compared to the mobility at 80% O_2_ (W = 300; p-value = 0.005). Under 21% O_2_, migration up to 19.5 cm was observed, but the median migration was only 0.3 cm in 30 min.

At 12 ℃, as shown in Table 1, more than 50% of *Anisakis* spp. larvae were motile. There was no significant difference of larval motility between the different O_2_ conditions (Fig. 4). For the mobility, the median migration of *Anisakis* spp. larvae was 0 cm at each of the O_2_ conditions and no significant differences of larval mobility were observed between different O_2_ concentrations (p > 0.05).

All *Pseudoterranova* spp. larvae at 6 ℃ and 12 ℃ were motile and there were no significant differences of larval motility between different O_2_ conditions at 6 ℃ and 12 ℃. For the mobility, at 6 ℃, the median migration of *Pseudoterranova* spp. larvae at the four different O_2_ concentrations ranged only from 0 to 0.3 cm in 30 min (see Additional file 2). At 12 ℃, similar as for *Anisakis* spp., there seemed to be a trend of higher larval motility and mobility at 21% O_2_, but the difference between the different O_2_ concentrations was not significant.

The number of larvae that are still motile over time until 6 h at different O_2_ conditions is shown in Table 2. After 6 h, there were very few *Anisakis* spp. larvae that were still motile, with five *Anisakis* spp. larvae being motile at 21% O_2_ while only one *Anisakis* spp. larvae was motile at 0% O_2_. More *Pseudoterranova* spp. larvae were motile after 6 h for all conditions with O_2_ (more than 55%) compared with *Pseudoterranova* spp. larvae at 0% O_2_ (only 20% larvae being motile).

## Discussion

The number of papers studying the effect of physical or chemical stimuli on movement of Anisakidae larvae is very limited. The few studies related to larval motility/mobility mainly focused on *post mortem* migration of larvae from viscera to fillets during long-term storage^25,26^ or aimed at killing the larvae^23^. The innovative aspect of this study was that it systematically investigated the short-term effect of temperature, CO_2_ and O_2_ on larval motility/mobility in vitro, under controlled conditions, aiming to identify a method that could trigger larvae to actively migrate out of fillets on the short-term. In this study, temperature was found to have a positive influence on larval movement. Cipriani et al. studied the response of *A. pegreffii* to the storage temperature of European anchovies after 24, 48 and 72 h (long term) and found that larval migration from the viscera to the fillets was positively related to the increase of fish storage temperatures from 2 °C to 5 and 7 ℃^26^. Cipriani et al. also found no statistically significant variation in infection either in the fillets or the viscera after 72 h when anchovies was stored at 2 ℃ indicating the importance of temperature on the *post mortem* motility of *A. pegreffii* larvae in anchovies^26^. In the present study, different temperatures had varying degrees of influence on larval movement. The movement of *Pseudoterranova* spp. larvae at 6 ℃ was significantly lower than the movement at other temperatures, which showed that low temperature could indeed inhibit the movement of larvae. Arthur et al. studied the effect of *post mortem* handling on larval abundance in the musculature and also found that storage of pollock on ice for 7 days did not result in any detectable migration of parasites from the body cavity into the musculature^37^. Also no loss of parasites from the musculature or significant change in the abundance of parasites in the musculature occurred^37^. However, in the study presented here, high temperatures such as 22 ℃ for *Anisakis* spp. larvae and 37 ℃ for *Pseudoterranova* spp. larvae, could significantly increase larval movement in a short time (5, 10, 30 min as investigated in this study). In fact, the mobility of *Anisakis* spp. larvae at 22 ℃ significantly differed from mobility at other temperatures already after 5 min. In the first 5 and 10 min, the median migration of *Anisakis* spp. larvae at 22 ℃ reached 3 cm and 10 cm respectively, which corresponds with several times the thickness of fillets. Therefore, high temperatures could increase larval movement on the short-term, which could facilitate larvae to actively move out of infected fish flesh. In this study, the most optimum temperature with maximum movement of *Anisakis* spp. larvae was 22 ℃, while the optimum temperature for *Pseudoterranova* spp. was 37 ℃. For *Anisakis* spp., larval mobility at 37 ℃ (1.7 cm) was already significantly decreased compared with mobility at 22 ℃ (30 cm). This could complicate using short-term storage at increased temperature in practice as many fish species were infected with mixed Anisakidae genera. According to Mehrdana et al*.* and Mercken et al*.*, simultaneous infection with *Anisakis simplex s. s.* and *Pseudoterranova decipiens* were recorded in cod^38^, haddock, plaice, pollack, whiting and gurnard^11^. On the other hand, for some fish species, such as salmon, halibut, leng, and dogfish, *Anisakis* spp. are normally more common than *Pseudoterranova* spp. larvae^11^. In addition, the median migration of *Pseudoterranova* spp. larvae at 22 °C was 4.5 cm in 30 min and 1.75 cm in 10 min, and an increased temperature of 22 ℃ already significantly increased the mobility of *Pseudoterranova* spp. larvae compared to lower temperatures. Short-term storage at 22 ℃ may thus be a promising method in practice for removing the infecting larvae.

For the effect of CO_2_ on larval movement at 6 ℃, there was no significant difference in larval migration between different CO_2_ concentrations in 30 min during which the migration of all larvae was very low. More long term effects were assessed at 6 ℃, with a longer observation of 1 min each 30 min until 6 h. After 6 h, only 8 out of 157 larvae (*Anisakis* spp. larvae and *Pseudoterranova* spp. larvae) were still mobile and the motility of larvae in the one-minute movies at the 6th hour were lower compared with larval motility in the first minute movies. In conclusion, CO_2_ did not have a significant effect on migration of both *Anisakis* spp. larvae and *Pseudoterranova* spp. larvae in 30 min and may thus not be applicable to trigger larvae to actively migrating out of fillets in an industrial setting either. At 12 ℃, there was no significant difference of larval motility and mobility found between different CO_2_ concentrations in 30 min both for *Anisakis* spp. larvae and *Pseudoterranova* spp. larvae. This means that a CO_2_ enriched atmosphere seems unsuitable in an industrial context to remove larvae from infected fishes on a short time (30 min). Although there was no meaningful impact of CO_2_ on larval mobility confirmed in 30 min and even 6 h, according to Pascual et al*.*, if several days of storage would be implemented, the total migration of *Anisakis* spp. larvae at 60% CO_2_ maybe observed higher than the total migration at other CO_2_ concentrations (Fig. 3)^20^. Pascual et al*.* studied the behavior of *Anisakis* spp. larvae in fish stored under different CO_2_ modified atmospheres at 3 °C. In that study, *Anisakis simplex *sensu stricto stored at 20% CO_2_ tended to a quiescence and mostly coiled behavior at day 15, whereas *Anisakis* spp. larvae stored at 55%-90% CO_2_ were active, stretched and larval spontaneous migration occurred after 15 days of storage. This can explain why in practice, an increased presence of visible active larvae on the surface of fish is observed when MAP is used at retail level^20^.

Under different O_2_ conditions at 6 and 12 ℃, both *Anisakis* spp. larvae and *Pseudoterranova* spp. larvae had no or only a very low median mobility. There is no meaningful impact of O_2_ on larval mobility and an O_2_ enriched atmosphere cannot be used in an industrial context to trigger larvae to actively move out of infected fillets.

The batch of larvae may also affect larval movement, as different factors (such as the origin and age of the larvae) may influence larval behavior. To account for this potential batch effect, in this study conditions were only compared within experiments that used larvae from the same batch(es). Also, we observed a high variability between larvae. For example, *Anisakis* spp. larvae from the same batch at 22 ℃ in the temperature experiment had a maximum migration of 74 cm and a minimum migration of only 7 cm in 30 min. This may be due to the different ages and physical conditions of larvae. The variability should be taken into consideration when assessing the practicability of the technique. Larval mobility in fish may be very different from larval mobility in PBS agar as reported in this study, because the composition and texture of PBS agar are different from fish. So validation of the temperature experiments in real fish fillets is necessary and a common temperature for both *Anisakis* spp. larvae and *Pseudoterranova* spp. larvae should be further investigated. Finally, the impact of a rapid, short term increase in temperature followed by a rapid decrease to the temperature of melting ice on other pathogens or spoilage bacteria should be investigated.

## Conclusions

In this study, the effect of temperature on larval movement of *Anisakis* spp. and *Pseudoterranova* spp. on a short-time exposure was clearly illustrated. Increasing temperature increased larval movement, but the optimal temperature for movement was different between the two investigated species. For *Anisakis* spp., the optimum temperature was around 22 ℃ whereas it was 37 ℃ for *Pseudoterranova* spp. Atmospheres enriched with CO_2_ or O_2_ could not facilitate larval movement significantly on a short-term. These results provide evidence for the effect of temperature on larval movements and could be a basis for further exploring a method for industrially removing larvae from infected fish with a short-term treatment.

## Supplementary Information

Supplementary Figure S1.

Supplementary Figure S2.

## Data Availability

All data are available, upon reasonable request, from the corresponding author.
