# Supplementary material for: Effect of temperature, CO2 and O2 on motility and mobility of Anisakidae larvae
Source: Sci Rep. 2021 Feb 19;11:4279. doi: 10.1038/s41598-021-83505-5 (PMC7895964; doi:10.1038/s41598-021-83505-5)
Supplement: Supplementary file 2 — Supplementary Figure S2. [file 41598_2021_83505_MOESM2_ESM.pdf]

Effect of temperature, CO_2 and O_2 on motility and mobility of Anisakidae larvae

Aiyan Guan[1,2,*], Inge Van Damme[2], Frank Devlieghere[1,•] and Sarah Gabriël[2,•,*]

Supplementary Figure 2

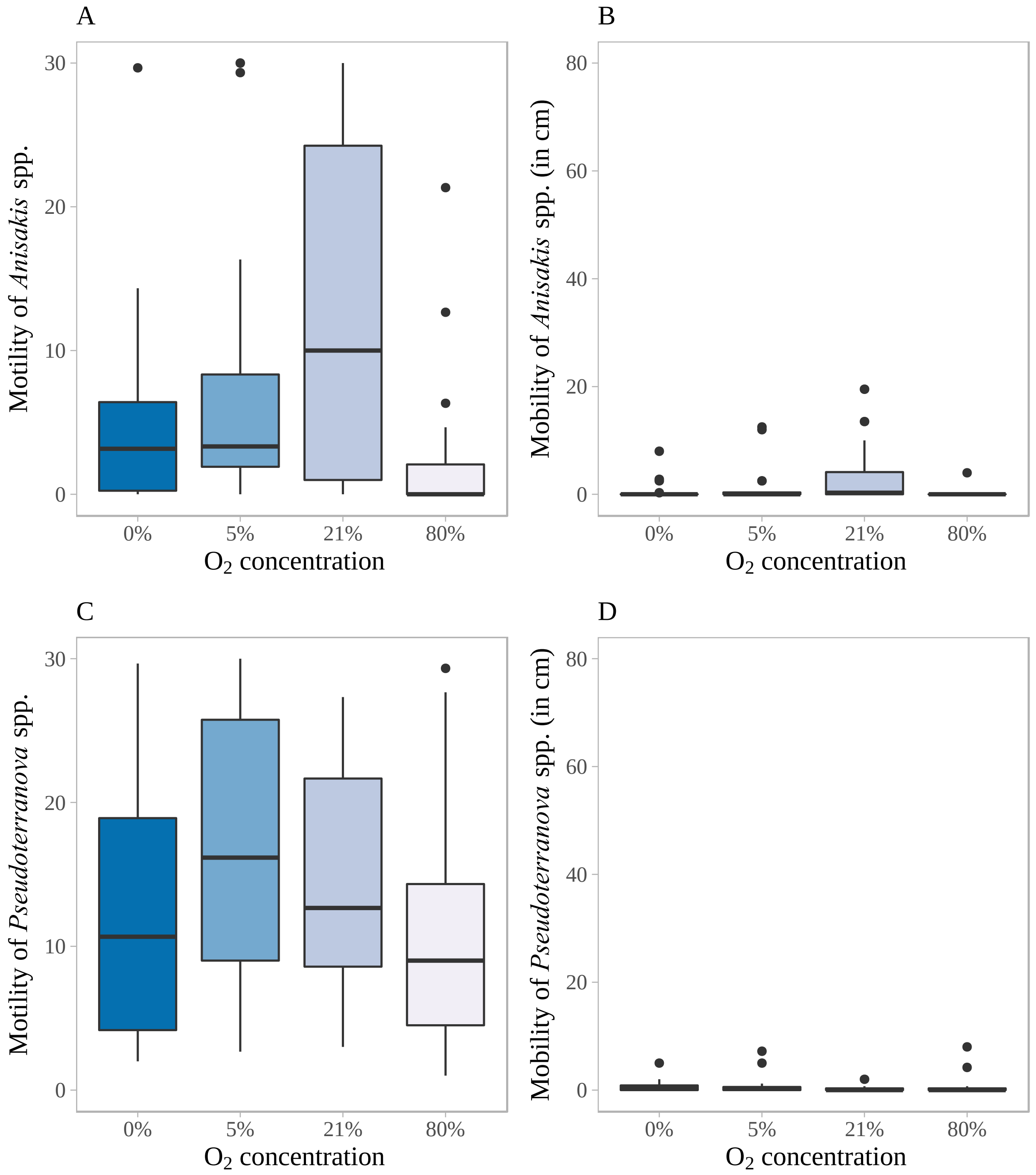

Supplementary Figure 2. Effect of O_2 on larval motility and mobility at 6 °C during the first 30 minutes. A: motility of *Anisakis* spp., B: mobility of *Anisakis* spp., C: motility of *Pseudoterranova* spp. and D: mobility of *Pseudoterranova* spp. under different O_2 conditions. The box consists of the upper quartile, median and lower quartile. The whiskers are drawn up to the highest or lowest observed point from the dataset that falls within 1.5 times the interquartile range. Observations beyond the end of the whiskers are plotted individually.
